# Qualitative Immune Modulation by Interleukin-2 (IL-2) Adjuvant Therapy in Immunological Non Responder HIV-Infected Patients

**DOI:** 10.1371/journal.pone.0014119

**Published:** 2010-11-29

**Authors:** Francesca Sabbatini, Alessandra Bandera, Giulio Ferrario, Daria Trabattoni, Giulia Marchetti, Fabio Franzetti, Mario Clerici, Andrea Gori

**Affiliations:** 1 Division of Infectious Diseases, Department of Internal Medicine, San Gerardo Hospital, University of Milan-Bicocca, Monza, Italy; 2 Department of Clinical Sciences, Infectious Diseases Section, Luigi Sacco Hospital, University of Milan, Milan, Italy; 3 Department of Preclinical Sciences, Chair of Immunology, LITA VIALBA, University of Milan, Milan, Italy; 4 Department of Medicine, Surgery and Dentistry, Clinic of Infectious Diseases, San Paolo Hospital, University of Milan, Milan, Italy; 5 Chair of Immunology, Milan University Medical School and Don C. Gnocchi Foundation IRCCS, Milan, Italy; University of California San Francisco, United States of America

## Abstract

**Background:**

Treatment of HIV-infected patients with interleukin-2 (IL-2) produces significant increases in CD4 T cell counts; however an associated qualitative improvement in cells function has yet to be conclusively demonstrated. By measuring mycobacterial killing activity, we evaluated IL-2-mediated functional immune enhancement *ex vivo* in *immunological non-responders* (INRs).

**Methods and Findings:**

PBMC from 12 immunological non-responders (INRs) (CD4+<200/µl, HIV-RNA<50 cp/ml) on combination antiretroviral treatment (cART) were collected at baseline, and after 3 IL-2 cycles. Eight INRs receiving only cART were studied as controls. After 21 days of PBMC incubation with a virulent *M. avium* suspension, counts of residual colony forming units (CFUs) and concentrations of TNF-α, IL-10 and IFN-γ were determined. In IL-2 treated patients, a significant reduction in mean residual CFUs of PBMC cultures was observed (p<0.01). Moreover, following IL-2 treatment, significant increases in PBMC's IFNγ production (p = 0.02) and substantial reductions in IL-10 levels were observed.

**Conclusions:**

IL-2 therapy restores the ability of the lympho-monocyte system in eliciting an effective response against mycobacterial infections. Our data indicate the possibility of a clinical role held by IL-2 in enhancing the immune function of subjects unable to achieve immune competence through cART alone.

## Introduction

Since its introduction into clinical practice, combination antiretroviral treatment (cART) has dramatically changed the course of HIV natural history, assuring optimal inhibition of HIV replication and leading to effective immune recovery [1]. Despite these advances, up to 40% of patients beginning cART with CD4+<50 cell/µL fail to achieve satisfactory CD4+ cells count levels, even after years of virologically effective antiretroviral treatment [2]. Among such individuals, *Immunological Non Responders* (INRs) stands out as CD4+ T cells in these patients remain consistently below 200 cells/µL [3], [4]. The underlying mechanism for this lack of immune response remains undiscovered. Persistent low CD4+ T cells count is a strong predictor of disease progression, death and development of non AIDS-related clinical events [5], and finding successful strategies for enhancing immune response in these patients is therefore essential. Given their particular problems in achieving satisfactory CD4+ levels despite cART, INRs are an intriguing target for immune-based approaches. Treatment of INRs with intermittent cycles of interleukin-2 (IL-2) was reported to result in significant increases in CD4+ T cells count [6]. By inducing a polyclonal expansion of CD4+ cells mainly characterized by a naïve or central-memory phenotype [7], IL-2 greatly enhances the proliferation and function of T-lymphocytes and natural killer cells and promotes the production and release of other cytokines [8]. IL-2 is particularly active on CD4+ T cells, both in enhancing proliferation of existing T cells and in temporarily boosting neothymopoiesis [9]. However, extended analysis of the qualitative features of these newly recovered cells is required in order to define the true functional advantages of IL-2 therapy. Recently, two randomized prospective studies, SILCAAT and ESPRIT [10], failed to demonstrate the clinical benefit of IL-2 adjuvant treatment. However, these trials were based on patients whose median CD4+ cells count were at least 200 cells per ml, compared to INRs who were the object of our own study. The aim of our study was to determine whether CD4+ cells gain induced by IL-2 in a population of INRs was also associated with qualitative and functional improvement of the immune performance. As a model for evaluating qualitative immune function, we measured killing activity on mycobacteria in peripheral blood mononuclear cells (PBMC) *ex-vivo*, from a group of INRs treated with intermittent cycles of IL-2. Reduced mycobacterial growth and enhanced production of certain cytokines involved in antigen specific response following IL-2 administration were considered to be signs of enhanced immune response.

## Methods

### Ethics Statement

Samples analyzed in our study derived from patients enrolled in an open-label randomized trial whose main results were published in 2002 [6]. HIV-infected immunological non responders enrolled in our study received effective antiretroviral treatment alone or in association with IL-2. The study was approved by institutional ethic committee of Institute of Infectious Diseases, Luigi Sacco, Milan. All individuals were enrolled by the Department of Infectious Diseases of the Luigi Sacco Hospital in Milan. Patients participating in this study gave written informed consent according to the Declaration of Helsinki.

### Study design and population

**Figure 1:**
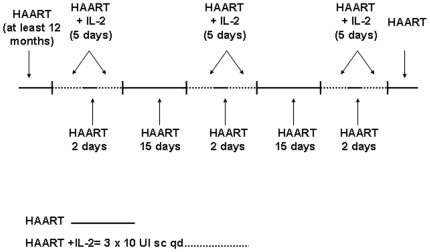
Schedule of IL-2 administration.

This study has been conducted selecting patients from 22 subjects enrolled in an open-label randomized Institutional Review Board-approved trial of IL-2 immunotherapy in association with cART versus cART alone, published by Marchetti *et al* [6]. The trial anticipated the ability of IL-2 adjuvant therapy of rescuing the CD4+ T cells compartment when used at low dose for a prolonged period. Twenty HIV-infected INRs patients were borrowed from the main trial and their samples analyzed. All subjects shared HIV RNA<50 copies/ml and CD4+ T cells count consistently <200 cells/µL after at least 6 months of stable cART. Twelve patients received recombinant human IL-2 (Proleukin; Chiron) administered at a dosage of 3×10^6^ IU daily as a single subcutaneous injection at days 1–5 and 8–12 of a 4 -week cycle, for a total of 3 cycles (overall, 10 weeks' duration), in addition to ongoing antiretroviral treatment (Fig. 1). The other 8 patients received cART alone. Persons who had previously been treated with IL-2 were excluded. Patients were evaluated at baseline and after three cycles of IL-2 and followed over 48 weeks. Peripheral blood mononuclear cells (PBMC) were obtained at baseline prior to IL-2 treatment (day 0: T0) and after 3 cycles of IL-2 therapy (day 75: T1) in IL-2 treated patients and at enrollment (day 0: T0) and after 75 days (day 75: T1) in cART-treated controls.

### Study Patients

Patients had an overall median CD4+ T cell count nadir of 53 cells/µL. Nadir were similar between the groups: median CD4+ cells number in treated patients was 54 cells (IQR, 50.5–80 cells/µL); median CD4+ cells number in controls was 38.5 (IQR, 18.75–54 cells/µL); *P* = 0.26. All patients received a combination antiretroviral regimen including either 2 nucleoside reverse-transcriptase inhibitors and 1 protease inhibitor or 1 non-nucleoside reverse-transcriptase inhibitor for a median time of 322 days (10.7 months) before enrolment; no differences were seen in length of antiretroviral treatment between IL-2 treated patients (median 322 days; IQR, 247–394 days) and controls, (median 310.5 days; IQR, 271–432.7; *P* = 0.72). Patients demonstrated sustained suppression of viral replication for at least 6 months before enrolment (HIV-1 RNA levels of <50 copies/mL in 3 consecutive determinations), and had CD4+ T cell counts consistently below the threshold of 200 cells/mL during cART. There were no baseline differences between the 2 groups with respect to age, epidemiologic data, CD4+ T cell counts, HIV RNA load, HIV-related parameters, or cART regimen at enrolment (Table 1). None of the patients reported opportunistic infections up to 1 year before study initiation. No patients had previous episodes of MAC infections, and therefore none were taking MAC primary or secondary prophylaxis.

**Table 1:** Baseline characteristics of patients enrolled in the study.

Characteristic	IL-2 treated group (N°12)	*Control group (N°8)*
**Male sex (%)**	8 (66.6%)	*6 (75%)*
**Median age [IQR]**	38 [33.5–41]	*42.5 [39.25–47.25]*
**Risk factors for HIV infection**		
**IVDU**	5	*5*
**MSM**	2	*2*
**Other**	5	*1*
**HIV RNA<50 copies (%)**	100	*100*
**CD4+ cell count nadir (per mm^3^)**		
**Median (IQR)**	54 [50.5–80]	*38.5 [18.75–54]*
**N° of opportunistic infections at baseline (%)**	0	*0*
**Patients on PCP profilaxis (%)**	100	*100*
**Patients on MAC profilaxis (%)**	0	*0*
**Current antiretroviral treatment (%)**		
**NNRTI+NRTI**	54.6	*25*
**PI+NRTI**	45.4	*75*
***Median number of days spent on antiretroviral treatment prior to enrollment [IQR]***	*322 [247–394]*	*310.5 [271–432.7]*

**IQR** Inter Quartile Range; **PI**: Protease Inhibitors; **NRTI**: Nucleoside Reverse Transcriptase Inhibitors; **NNRTI**: Non-Nucleoside Reverse Transcriptase Inhibitors.

### *Mycobacterium avium* and inoculation of PBMC cultures

A virulent strain of *M. avium* was isolated and identified from a clinical sample of a patient affected by *Mycobacterium avium* disseminated infection [11], [12]. For inoculation of PBMC cultures, an *M. avium* stock aliquot was thawed and suspended in isotonic saline. The *M. avium* suspension was homogenized and sonicated in a sonicating waterbath for 3 min at room temperature to disrupt mycobacterial clumps. Mycobacterial concentration was determined through optical density (OD) and adjusted to 3×10^7^/ml. The *M. avium* suspension was serially diluted 10-fold and triplicate 10-µl spots of the dilutions were plated on agar medium (Middlebrook 7H10 plus 10% OADC, Becton Dickinson, USA) to verify the number of organisms per milliliter of the inoculation suspension [13]. The mycobacterial suspension was utilized to inoculate 1- and 3-×10^6^ cells/well PBMC cultures, adding 33- and 100-µl of the suspension/well respectively, at a ratio of 1 organism per cell. For each donor, PBMC cultures were prepared in order to allow for the following tests in duplicate, at T0 and T1: PBMC at 1- and 3-×10^6^ cells/well (2 ml culture medium volume); *M. avium*-inoculated PBMC at 1- and 3-×10^6^ cells/well (2 ml culture medium volume), mycobacteria/cells ratio being 1∶1. Cultures were incubated at 37°C and 5% CO_2_, for as long as 21 days, the mean time for mycobacterial replication. Cell count and viability were assessed by trypan blue exclusion dye assay at the conclusion of tests, using control cultures; cell typing of residual cells was also performed by flow cytometry (murine mAb anti-human CD14 by Coulter Electronics Inc., Miami Lakes, FL, USA and Epics Elite cytometer by Coulter Electronics) at the conclusion of tests, using control cultures.

### Evaluation of mycobacterial growth by CFU counting

After 21-days incubation, *M. avium*-infected macrophages were lysed by SDS (final concentration 0.1%) for 15 min at room temperature. The SDS was neutralized by adding an equal volume of 20% bovine serum albumin to each well and cell lysates were flushed repeatedly with an automatic pipette. The cell lysates were harvested, sonicated for 20 seconds, serially diluted 10-fold, and plated on Middlebrook 7H10 plus OADC agar in 6-cm petri dishes (three 10-µl spots were plated per dilution). The agar plates were incubated for 28 days at 37°C/5% CO_2_ and individual *M. avium* colonies were enumerated with an inverted microscope after 14 and 28 days of incubation.

### Cytokine Elisa assays

Because of their ascertain role in immune activation against mycobateria, we evaluated supernatant concentration of TNF-α, IFN-γ and IL-10 from *M. avium*- infected PBMC cultures. Supernatants (500µL) were harvested and replaced with fresh culture medium at day 0 (immediately before *M. avium* inoculation), at day 7, day 14 and at day 21 (immediately before lysing of infected cultures). Supernatant samples were stored at −20°C. Cytokine concentrations was performed as per manufacturer's instructions using commercial kits (Endogen Human Elisa, Endogen, Inc., Woburn, MA, USA; detection limit of 5 pg/ml).

### Statistical analysis

PBMC cultures from different donors varied as to *M. avium* growth. For this reason, number of surviving bacteria were calculated both as absolute value and as percentage variation. Results were expressed as median number of colony-forming-units (CFU) and Inter Quartile Range (IQR) per milliliter of lysate of each experimental condition. Cytokine concentrations were expressed as median pg value and IQR per milliliter of each experimental condition. Mann-Whitney test and Student's paired and unpaired *t*-test were performed in order to determine the statistical significance of differences in *M. avium* growth and cytokines concentrations. Correlation analysis was made using a non-parametric test.

## Results

### Effects of IL-2 treatment on *M. avium* growth

**Figure 2:**
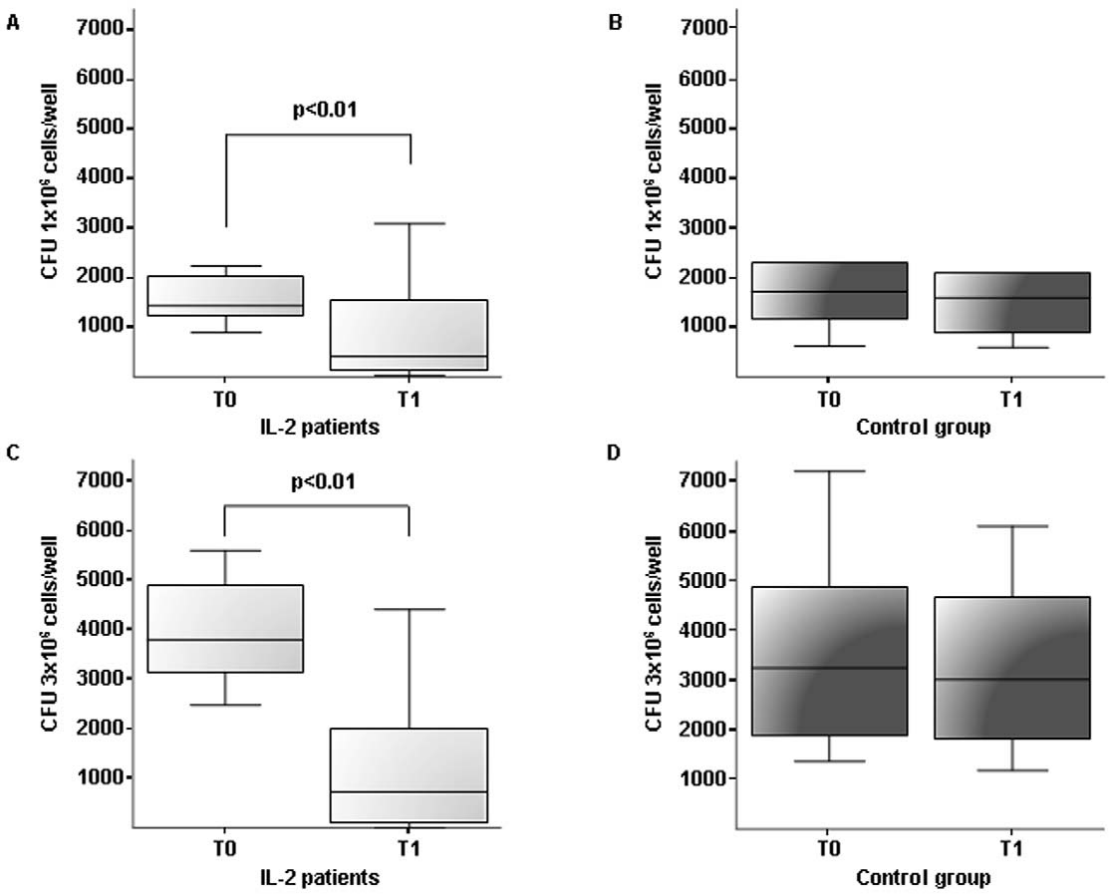
Mycobacterial growth (CFU: Colony Forming Unit) before (T0) and after (T1) IL-2 adjuvant treatment (A,C) compared to cART treatment alone in control group (B,D). CFU growth has been tested in different aliquots of PBMC cultures of 1×10^6^ cells/well (A,B) and 3×10^6^ cells/well (C,D). In IL-2 treated patients a significant reduction in mean residual mycobacterial growth was observed in both experimental conditions of PBMC cultures (*P*<0.01). Significant results are emphasized by square brackets with respective *P* values.

*M. avium* was present in all PBMC cultures and residual CFUs produced colonies primarily of flat, transparent type. Analysis of 1-×10^6^ cells/well cultures in IL-2 treated patients resulted in a median number of CFU/mL of 1450 (IQR, 1245–2035) at T0 and of 420 (IQR, 135–1545) at T1 (*P* = 0.008). Control patients displayed a median CFU number of 1725 (IQR, 1247–2202.5) at T0 and of 1590 (IQR, 1025–2020) at T1 (*P* = 0.16). In the second experiment, using 3-×10^6^ cells/well, the median number of CFU was 3340 (IQR, 3167.5–4800) at T0 and 670 (IQR, 260–1782.5) at T1 in IL-treated patients, (*P* = 0.001), while in PBMC from patients receiving only cART, median CFU number was 3220 (IQR, 2105–4552.5) at T0 and 3015 (IQR, 2037.5–4345) at T1 (*P* = 0.106); (Fig. 2). This significant reduction in mycobacterial growth suggests an improved killing activity of PBMC after IL-2 treatment.

### Effects of IL-2 treatment on cytokines production

**Figure 3:**
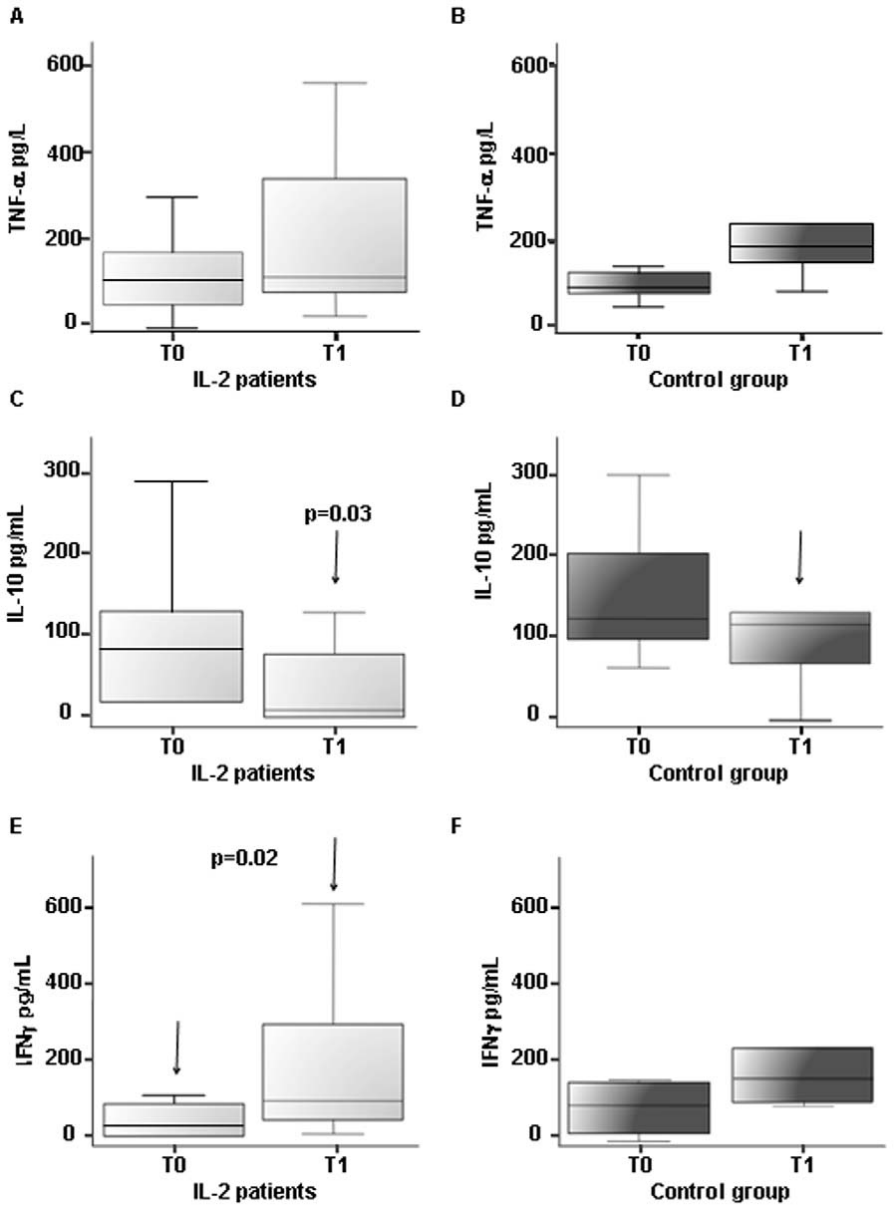
Production of TNF-α (A,B), IL-10 (C,D) and IFN-γ (E,F) in PBMC cultures before (T0) and after (T1) IL-2 adjuvant treatment (A,C,E) compared to cART treatment alone in control group (B,D,F). Following IL-2 treatment significant increases in PBMC's IFN-γ production (*P* = 0.02), and substantial reduction in IL-10 levels were observed resulting in significant difference between IL-2 patients and controls at T1 (*P* = 0.03). Significant result in IL-2 treated patients are emphasized by black arrows indicating the compared groups with respective *P* value.

Supernatant concentrations of cytokines IL-10, TNF-α and IFN-γ were determined by ELISA assay on samples harvested at days 0, 7, 14 and 21 from inoculation. Cytokines release by PBMC with culture medium alone was virtually undetectable in every donor group at each determination performed before day 21 (days 0, 7, 14; data not shown). No significant differences were observed in TNF-α concentrations at T1, when comparing treated patients to controls. Likewise, at day 21, non significant variation in TNF-α concentrations from T0 to T1 was observed, in both IL-2 treated patients (T0 median value 111 pg/ml; IQR, 59.5–165.8; T1 median value 116 pg/ml; IQR, 83–307.9; *P* = 0.07), and controls (T0 median value 94 pg/ml; IQR 78.5–117.8; T1 median value 191 pg/ml; IQR, 159.8–235.4; *P* = 0.06) (Fig. 3 A,B). IL-2 treated patients presented a reduction in IL-10 concentrations (T0 median value 79 pg/ml; IQR, 66–123.2; T1 median value 8 pg/ml; IQR, 65.4–110), whereas no changes were shown in controls (T0 median value 121 pg/ml; IQR 107.1–161.9; T1 median value 119 pg/ml; IQR, 81.2–132.3) resulting in significantly different IL-10 levels at T1 between the two groups (*P* = 0.03; Fig. 3 C,D). Finally, IFN-γ production substantially increased in IL-2 treated patients between T0 and T1 (T0 median value 18 pg/ml; IQR, 31.2–67; T1 median value 87 pg/ml; IQR, 38.6–248; *P* = 0.02) whereas no significant changes were observed in controls between the two time points (T0 median value 85 pg/ml; IQR, 38.5–124.82; T1 median value 163 pg/ml; IQR, 116.23–228.6; *P* = 0.13) (Fig. 3 E,F).

### Correlation between mycobaterial killing and cytokines production

To evaluate if a direct link between the effects of IL-2 treatment on cytokines production and mycobacterial killing activity exists, we investigated if in IL-2 treated patients and controls MAC growth correlates with levels of IL-10, TNF-α and IFN-γ. In IL-2 treated patients a significant negative correlation was seen between *Mycobaterium avium* CFU and TNF-α concentration. (r = −0.89, *P* = 0.02), thus revealing a possible connection between the production of this cytokine and the improved killing activity. Moreover, in IL-2 treated patients IL-10 concentrations were inversely associated with TNF- α levels (r = −0.89, *P* = 0.02), whether a direct correlation was found between IFN- γ and TNF-α production (r = 0.97, *P* = 0.001).

## Discussion

We explored the role of IL-2 treatment in improving the functional ability of lympho-monocytes in deeply immunocompromised patients with HIV infection. Accordingly to previous findings of the main trial [6], our data show that IL-2 adjuvant treatment is effective not only in raising CD4+ cells in number, but also in enhancing the specific immune system functional properties. In particular results herein show that IL-2 is associated with increased mycobacterial killing possibly due to inducing cytokine release and synthesis. Up to 44% of patients starting cART with very low CD4+ cells number are unable to reach a CD4 cell count >500 cells/µ despite years of virological suppression [2]. Several studies demonstrate that INRs, when compared to patients who fully respond to antiretrovirals [14], are prone to a higher incidence of AIDS defining illnesses or other opportunistic events typical of clinical progression [15], [16]. Effective strategies aimed at achieving improved immunological status for these patients [17] are required, and several immunomodulant approaches, including cytokines able to stimulate cell-mediated immunity, have been attempted [18], [19]. Previous studies have demonstrated that IL-2 effects a significant, sustained and selective rise in CD4+ -T cells number by stimulating CD4+ cells neothymic synthesis, redistribution of central and effector memory T cells and improving the functionality of such cells. This suggests a possible role of this cytokine in selectively reconstituting T helper activity to neoantigens [20], [11]. Nevertheless, results of two randomized prospective studies in HIV positive patients with CD4+>200 cell/µL [10], recently failed to demonstrate clinical benefits in IL-2 treated patients compared to cART alone; in fact, IL-2 treated subjects and controls experienced a similar rate of opportunistic diseases and deaths. However, INRs population was not specifically targeted in either of these trials. On the contrary, both studies involved patients where median CD4+ cells count was at least 202 cells per mm^3^, significantly higher than in our study (mean CD4 cells nadir: 56,7 cells/mm^3^). Notably, the number of AIDS and non-AIDS related events is significantly lower in persons with better immunological status, such as the patients enrolled in SILCAAT and ESPRIT. Therefore, while these trials indeed confirm the ability of IL-2 to induce sustained quantitative gains in CD4+ cells, they fail to find a clinical advantage to the growth of these new cells, leaving unresolved the question of their properties. Nevertheless, SILCAAT and ESPRIT did provide a promising hypothesis: that IL-2 may enhance proliferation of T cells pools which are different from those expanded by antiretroviral therapy alone and characterized by naïve and central memory phenotypes. We attempted to demonstrate if indeed this higher number of cells was accompanied by a qualitative improvement in immune function. To this end, we tested the quality of the immune response by evaluating the *ex vivo* mycobacterial killing capacity of IL-2 induced lympho-monocyte cells in a population of INRs treated with IL-2. Previously, several authors [21], [22] have shown that *Mycobacterium avium* killing is defective in cells of HIV-infected patients [23]. Herein we show that such defect can be corrected with IL-2 administration. In particular IL-2 appears to restore the ability of the lympho-monocyte system in mounting an effective defence against mycobacterial infections enhancing killing activity. Interestingly, the improved killing activity parallels an increased release of specific cytokines, whose production is altered during HIV infection. Following IL-2 treatment we detected decreased levels of IL-10, a macrophage-suppressive cytokine. Furthermore, IL-2 administration in our study resulted in higher concentrations of IFN-γ, a key mediator of bactericidal activity of macrophages. Inhibition of the release of cytokines such as IFN-γ is in fact one of the classic IL-10 functions [24]. Our data suggest a potential link between macrophage activation and IL-2-induced modulation of cytokines release. These results reflect the complexity of the immune response to mycobacterial infections induced by IL-2, in which Th1 cytokines such as IFN- γ function as activators of macrophages, thereby promoting intracellular killing. In the context of mycobacterial infection, IL-10 reduction enhances actions of stimulatory cytokines contributing to host cells defence [25]. Our findings indicates that *in vivo* IL-2 therapy results in a beneficial immune modulation in individuals in whom cART does not significantly improve CD4 counts. Because of the higher risk of developing opportunistic infections or AIDS and non AIDS-related events, patients with very low immunologic competences may benefit from IL-2 adjuvant treatment more than patients who fully respond to antiretrovirals. Careful identification of target patients could offer a supplementary opportunity for immunological improvement in particular kinds of HIV- infected subjects.
